# A Metachronous, Atypical, Multifocal Renal Oncocytoma with a Concomitant Renal Cell Carcinoma of the Contralateral Side and Bilateral Multifocal Oncocytomas: Two Case Reports and Review of Literature

**DOI:** 10.1100/tsw.2005.73

**Published:** 2005-07-20

**Authors:** Maximilian Burger, Stefan Denzinger, Thomas Filbeck, Arndt Hartmann, Wolfgang Rößler, Christina Hammerschmied

**Affiliations:** ^1^ Department of Urology, University of Regensburg, Germany; ^2^ Department of Pathology, University of Regensburg, 93042 Regensburg, Germany

**Keywords:** kidney tumors, oncocytoma, renal cell carcinoma

## Abstract

We present one case of a metachronous, atypical, multifocal renal oncocytoma with a concomitant chromophobe renal cell carcinoma (RCC) of the contralateral side and one case of bilateral and multifocal oncocytomas. Oncocytomas are benign renal tumours that rarely appear bilateral or multifocal or with coexisting RCC. A common pathogenic denominator of oncoytomas and RCC is being discussed. The first case was a 63 years old patient presenting with a history of nephrectomy for a pT1 G1 pN0 R0 papillary RCC 4 years prior to presentation, showed two tumours of a singular kidney. Upon nephron-sparing surgery one typical and one atypical oncocytoma with an invasion of the perinephric fat were found. Comparative genomic hybridisation was performed. Both tumours revealed genetic alterations with loss of genetic material on chromosome 1p. The second case was a 62 years old patient presenting with multifocal and bilateral renal tumours of undeclared dignity upon imaging. During open exploration all tumours could be removed by nephron-sparing surgery and were identified as oncocytomas. Again comparative genomic hybridisation was performed. All 4 tumours revealed genetic alterations with loss of genetic material on chromosome 1p, one of the tumours an additional loss of chromosome 10.

## INTRODUCTION

Oncocytomas are benign epithelial tumors of the kidney. Since clinical differential diagnosis from RCC is difficult, most patients undergo surgery. Oncocytomas usually appear to be unifocal, but multifocal and bilateral appearance and concomitant RCC have been reported. The histopathological differentiation is not always obvious and a pathogenetic relation with RCC is discussed.

## CASE 1

A 63-year-old patient presented with an incidental tumor of the left kidney. Radiological imaging by CT-scan revealed a 6- × 6-cm mass with homogenous uptake of contrast imposing as malign. The contralateral kidney showed a 1.5-cm, hyperdense mass adjacent to the lateral middle section of mild contrast uptake, which could not be evaluated; a nodal process was contemplated. Family history was negative concerning renal neoplasia; no pathological findings including skin lesions were found on physical examination. The left kidney was surgically exposed and nephrectomy was performed. Histopathology revealed a papillary RCC (pT1 G1 pN0). A CT-scan 13 months later showed the lesion of the left kidney progressing in size to 1.7 cm and an additional dorsal mass of the lower section of the kidney of 1 cm. Further progression in size to 3 and 2.5 cm, respectively, was noted by CT-scan 18 months later. Evaluation by MRI revealed two 3-cm masses with hyperperfusion on contrast. Due to the uncertain dignity, the kidney was then exposed and the two tumors enucleated.

Histopathology revealed two tumors with microscopic features of oncocytomas. The upper tumor showed one focal infiltration of the fat capsule. Immunohistochemistry demonstrated positive staining with anticytoceratin 7 antibody and the antimitochondrial antibody 113-1; negative staining with anticytoceratin 20 antibody was noted. Thus, the lesions were recognized as one typical and one atypical oncocytoma. Comparative genomic hybridization was performed using standard methods[1]. Both investigated tumors revealed identical genetic alterations with loss of genetic material on chromosome 1p. No additional genetic alterations were found. Follow-up concerning the oncocytoma so far is 18 months and 4 years after concerning RCC, respectively; the patient remains free of disease.

## CASE 2

A 62-year-old patient presented with bilateral tumors of the kidney; a CT-scan showed two lesions of the left kidney, each 3.5 cm in diameter, and two masses of 2- and 1.5-cm diameter of the right kidney (Fig. 1). All defied unanimous classification. Renal angiography demonstrated two hypervascularized, well-circumscribed tumors in each kidney (Fig. 2). Family history was uneventful for any neoplasia, physical examination did not demonstrate any further pathological feature, and no skin abnormalities were noted. Due to the uncertain dignity, both right-sided tumors were removed by enucleation. Frozen section demonstrated oncocytoma in both lesions. Both contralateral tumors were enucleated 1 week later. Frozen section again demonstrated oncocytomas. Definite histopathology demonstrated renal oncocytoma in all four tumors. Comparative genomic hybridization was performed using standard methods[1]. All four investigated tumors revealed identical genetic alterations with loss of genetic material on chromosome 1p. One of the tumors showed in addition loss of chromosome 10. No additional genetic alterations were found. At 24-month follow-up, the patient shows no progression.

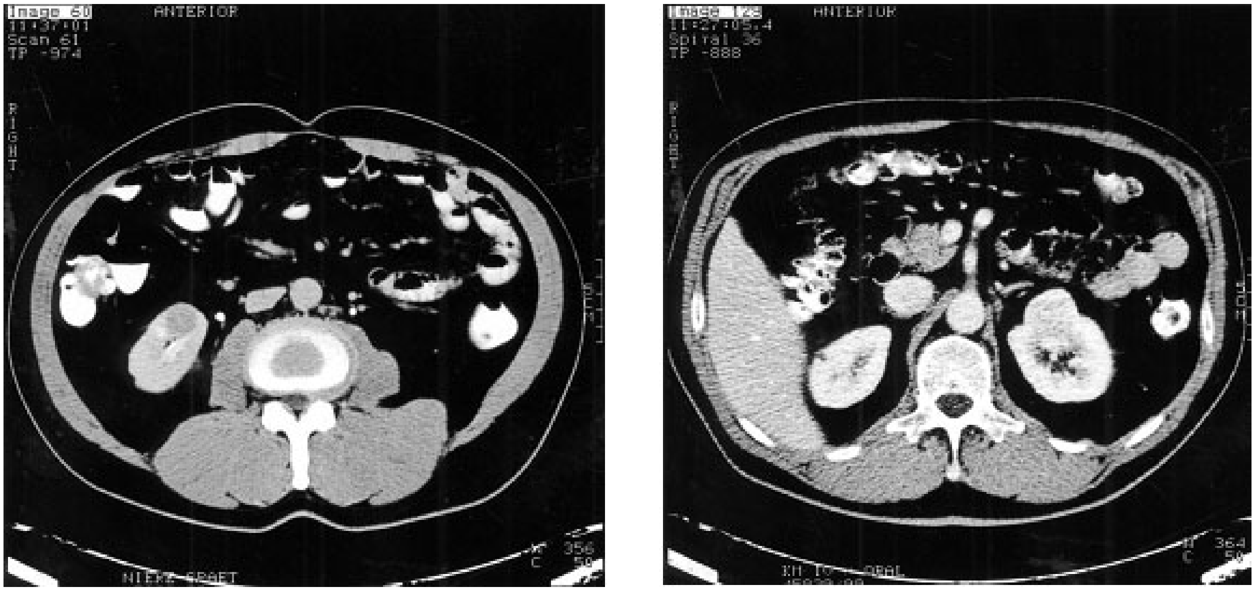

**FIGURE 1.**

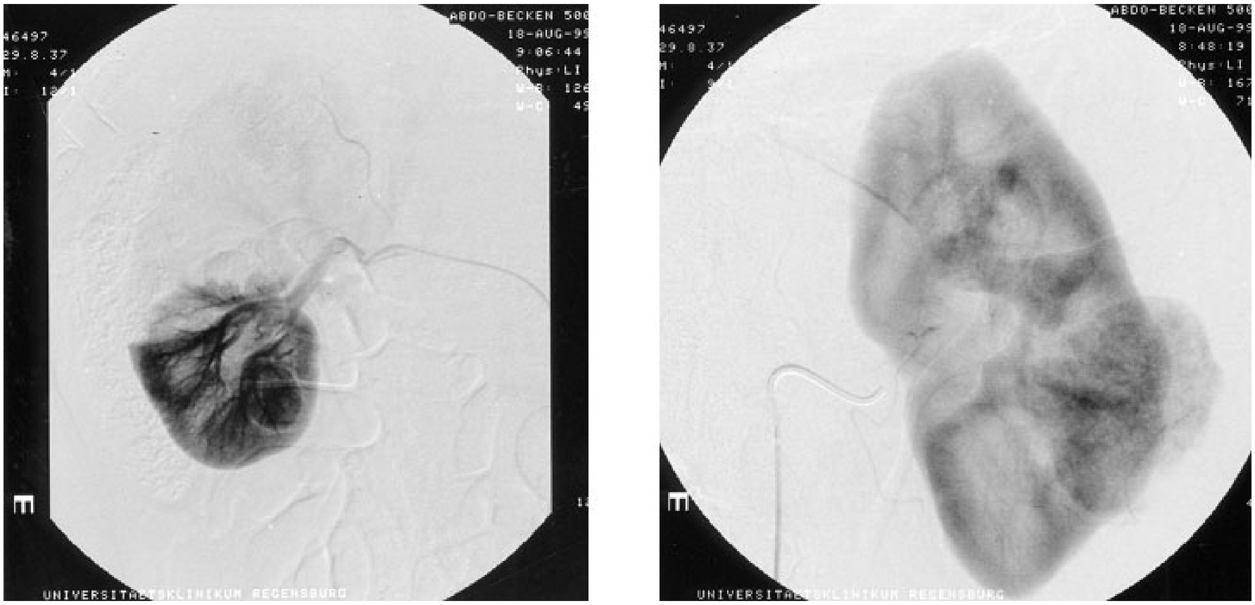

**FIGURE 2.**

## DISCUSSION

Renal oncocytomas comprise about 3–10% of all neoplasms of the kidney and are detected by chance mostly. They usually appear as solitary lesions measuring 4–8 cm. Multifocal and bilateral appearance have been observed in 4–6% and 4%, respectively[2,3]. Accordingly, a multifocal and bilateral growth pattern is rare. As observed in our cases, about 4% feature metachronous appearance and up to 10% concomitant RCC, respectively[3].

The oncocytic cell type expresses electronmicroscopical and immunohistochemical characteristics of the intercalated cell type A of the collecting duct and usually grows in an acinar pattern[4]; oncocytome are regarded as benign[2,3,5]. As in the cases presented here, histological differentiation from RCC can be challenging. The demonstration of cytoceratin 7 and of the mitochondrial antigen 113-1 would hint at RCC; expression of cytoceratin 20 would hint at urothelial neoplasm[6]. Specific genetic alterations can be helpful in distinguishing RCC from oncocytoma[7,8]. The oncocytic tumors described in this study show the typical deletions of chromosome 1p.

Some oncocytomas demonstrate malign phenomena. Some rare foci show an infiltration of the perinephric fat. Even metastatic disease has been reported[2]. However, it is possible that an eosinophilic RCC has been misdiagnosed for an oncocytoma in some cases[3]. Heterogeneous tumors featuring oncocytoma- and RCC-like areas have been described as well. This form is sometimes seen as the linking part of a spectrum, with the typical oncocytoma and the chromophobe RCC forming the extremes[9]. Genetic evaluation shows a common tendency towards the loss of certain chromosomes and an oncogenetic pathway from oncocytomas with aberrant chromosomes to RCC has been suspected[8,10].

No safe preoperative diagnostic differentiation between oncocytomas and RCC can be achieved. Angiography sometimes reveals the typical spoke-wheel formation; CT-scans show oncocytomas to be more homogenous than RCC, but especially small malign findings can be of a homogenous appearance. No differentiation using MRI is possible[5]. Bioptic procedures contain the risk of seeding tumor cells and cannot establish a safe distinction as well[11].

Bilateral renal tumors pose a special diagnostic and therapeutic challenge. Since a coexistence of oncocytomas and RCC evading even advanced imaging techniques cannot be excluded preoperatively[11], techniques such as cryotherapy and radioablation are rejected and a surgical exploration seems warranted[3,5]. A radical excision seems important, as recurrent disease is possible, especially if numerous small foci are present[12]. This feature has been termed *oncocytomatosis*.

In the cases presented here, RCC was hypothesized and a surgical approach chosen. Nephron-sparing surgery should be sought after, since metachronous development is possible. In one of the cases presented here, further lesions developed and the patient had to undergo further surgery. Due to this metachronous time pattern, follow-up examinations are warranted.

## CONCLUSION

Since unanimous preoperative diagnosis of an oncocytoma and reliable distinction from a RCC is rare, surgical exploration should be sought after for all uncertain cases. Due to metachronous appearance leading to further surgery, a nephron-sparing approach should be chosen. The metachronous time pattern demands follow-up examinations.
